# TOPBP1 as a potential predictive biomarker for enhanced combinatorial efficacy of olaparib and AZD6738 in PDAC

**DOI:** 10.1186/s13578-025-01350-9

**Published:** 2025-02-07

**Authors:** Xiao-Mei Tang, Min-Min Shi, Jia-Cheng Wang, Yi-Jin Gu, Yu-Ting Dai, Qin-Xin Yang, Jia Liu, Ling-Jie Ren, Xin-Yun Liu, Chun Yang, Fang-Fang Ma, Ji-Bing Liu, Hong Yu, Da Fu, Yun-Feng Wang

**Affiliations:** 1 Department of General Surgery, Pancreatic Disease Center, Ruijin Hospital, Shanghai Jiaotong University School of Medicine, Shanghai, 200025 China; 2 Institute of Oncology, Affiliated Tumor Hospital of Nantong University, Nantong, 226631 Jiangsu China; 3 Research Institute of Pancreatic Diseases, Shanghai Key Laboratory of Translational Research for Pancreatic Neoplasms, Shanghai Jiaotong University School of Medicine, Shanghai, 200025 China; 4 Shanghai Pinghe School, Shanghai, 200127 China; 5 Department of General Surgery, Pudong New Area People’s Hospital, Shanghai, 201299 China; 6 Department of Pathology, Ruijin Hospital, Shanghai Jiao Tong University School of Medicine, Shanghai, 200025 China; 7 State Key Laboratory of Oncogenes and Related Genes, Institute of Translational Medicine, Shanghai Jiaotong University, Shanghai, 200025 China; 8 Department of Pathology, The Affiliated Taizhou Peoples Hospital of Nanjing Medical University, Taizhou, 225300 Jiangsu China; 9 Department of Anesthesiology, The Affiliated Taizhou People’s Hospital of Nanjing Medical University, Taizhou, 225300 Jiangsu China; 10 Department of Pathology, Taizhou School of Clinical Medicine, Nanjing Medical University, Taizhou, 225300 Jiangsu China

**Keywords:** PDAC, TOPBP1, Olaparib, AZD6738

## Abstract

Pancreatic ductal adenocarcinoma (PDAC) is a highly aggressive and often lethal malignancy, requiring the development of enhanced therapeutic approaches. The DNA damage response (DDR) pathway is frequently altered during PDAC development, leading to an increased occurrence of DNA damage. DNA topoisomerase II-binding protein 1 (TOPBP1) plays a supportive role in regulating the DDR pathway, and its overexpression has been linked to the tumorigenesis of various cancers. This study investigated the biological role of TOPBP1 in PDAC pathogenesis and evaluated its clinical relevance in guiding treatment regimens. We examined the relationship between *TOPBP1* expression, DDR pathway modulation, and therapeutic response in PDAC cell lines, primary cells, and subcutaneous mouse models. We found that elevated *TOPBP1* expression was positively correlated with increased histologic grade and reduced patient survival in PDAC. *TOPBP1* knockdown increased the sensitivity of PDAC cells to olaparib treatment and improved therapeutic efficacy in both PDAC cell lines and subcutaneous mouse models. Combination treatment with olaparib and AZD6738 effectively induced P53-dependent apoptosis via inhibiting the ATR pathway and enhancing signaling through the ATM pathway, which significantly reduced the viability of pancreatic cell lines. Notably, this combination therapy was more effective in PDAC cell lines exhibiting high *TOPBP1* expression, indicating that TOPBP1 may serve as a useful predictive biomarker. In conclusion, TOPBP1 is a potential marker for optimizing the olaparib and AZD6738 combination therapy in PDAC. This study highlights the clinical significance of TOPBP1 in the treatment of PDAC and emphasizes the potential implications for a broader population of patients.

**Supplementary Information:**

The online version contains supplementary material available at 10.1186/s13578-025-01350-9.

## Introduction

Pancreatic ductal adenocarcinoma (PDAC) is a deadly solid tumor with a steadily rising incidence, and is expected to become the second most common cause of cancer-related mortality by 2030 [1]. Unfortunately, current treatment options, with radical resection as the primary approach, are often ineffective. Although combination chemotherapy, including folic acid, gemcitabine/albumin paclitaxel [2], and nanoliposomal irinotecan/fluorouracil, has been utilized as a first-line regimen, it only provides a survival advantage of 2–6 months compared to gemcitabine alone, with a median survival duration of 8.5–11.1 months [3]. Accordingly, it is imperative to develop novel combination regimens and targeted treatment to expand the patient population who can benefit from PDAC treatments [4, 5].

TOPBP1 (DNA topoisomerase II binding protein 1) is a binding protein comprising eight BRCT (BRCT1–BRCT8) and one ATR-activating structural domains [6–9]. It plays a critical role in various cellular processes, including DNA replication, DNA damage repair (DDR), and cell cycle checkpoints [10–13]. In particular, extensive research has focused on the role of TOPBP1 in DDR [10, 14, 15]. Notably, TOPBP1 has also been associated with tumorigenesis in multiple cancer types (Fig. S1A, S1B). However, further investigation is necessary to elucidate the complex role of TOPBP1 in the etiology and DDR of pancreatic cancer, as well as its clinical significance.

PDAC is characterized by complex rearrangements and mitotic abnormalities, with frequent changes in the DDR pathways, including homologous recombination (HR) abnormalities in the breast cancer 1 (BRCA1), breast cancer 2 (BRCA2), Ataxia Telangiectasia Mutated (ATM) and Partner and Localizer of BRCA2 (PALB2) genes [16–18]. The DDR pathway is a complex system developed to safeguard cells against acquired genome alterations and intrinsic and extrinsic DNA damage [19, 20]. Although various processes, including base excision repair (BER), nucleotide excision repair (NER), HR, and non-homologous end joining (NHEJ), are employed to repair DNA damage, their dysregulation can result in DNA damage accumulation, eventually leading to cancer formation, including PDAC.

The ATM and ATR pathways are essential in various aspects of the DDR. The ATM kinase is activated by DNA double-strand breaks, which cause chromatin alterations and trigger the G1 checkpoint by activating checkpoint kinase 2 (CHEK2) [21]. Similarly, the ATR kinase is activated by DNA double-strand breaks and inhibited transcription, leading to activation of the intra-S checkpoint. ATR then stimulates checkpoint kinase 1 (CHEK1), resulting in CDC25A degradation and cessation of the cell cycle's progression through S phase. These two pathways are upstream of the DDR pathway, and thus, significantly influence the direction of the DDR pathway [22].

Deficient DDR may enhance the susceptibility of PDAC to therapeutic interventions that exceed the tolerable threshold for DNA damage [23]. Poly (ADP-ribose) polymerase (PARP) inhibitors [24] are one such intervention capable of inducing DNA damage. And multiple synthetic lethal connections have been identified among DDR genes. Clinically, PARP inhibition has been used to target malignancies. For instance, the FDA has approved olaparib—a PARP inhibitor—for treating PDAC in patients with *BRAC1/2* mutations or HR abnormalities [25]. This has significantly increased the progression-free survival after platinum-based treatment. However, *BRCA1* or *BRCA2* mutations [26, 27] are present in a small subset of PDAC cases (only 5–7%). Meanwhile, 10–15% of PDAC cases have other DDR abnormalities, besides having mutated BRCA. Hence, new therapeutics targeting the DDR systems and cell cycling, such as ATR and WEE1 inhibitors, have also been explored [28–31]. To broaden the indications for PARP inhibitors and other DDR inhibitors and explore targeted drugs for PDAC, an in-depth analysis of the complex regulatory relationships within the DDR pathway is warranted [32, 33].

This study investigates the biological role and clinical significance of TOPBP1 as a marker of PDAC pathogenesis and DDR pathway. The associated findings expand upon those of previous studies on TOPBP1 and DDR mechanisms, supporting the use of DDR inhibitors for a wider range of indications [14, 32].

## Materials and methods

### Cell lines

The cell lines: Patu8988, BXPC3, AsPC-1, PANC-1, CFPAC, and MIAPaCa-2, were sourced from the Cell Bank of the Chinese Academy of Sciences in Shanghai, China. Each cell line was cultured in accordance with the manufacturer's instructions. Additionally, primary cell lines (0001, 0037, 0049 [34]) derived from PDAC patients' tumor tissues were established, maintained, and verified by WuXi Apptec Co. (Shanghai, China) and cultured in accordance with the supplier's protocol.

### Inhibitors

The PARP inhibitor, olaparib (Selleckchem, Houston, TX, catalog no. S1060) and the ATR inhibitor, AZD6738 (Selleckchem, Houston, TX, catalog no. S7693) were procured from indicated vendors. Olaparib stock solutions were prepared in DMSO at concentration of 20 mmol/L. AZD6738 stock solutions were prepared in DMSO at concentration of 10 mmol/L.

Construction of *TOBP1-*knockdown cells.

The shRNA sequences used in this study were: shTOPBP1#1 (5′-GCCUUUACAUGAUUCAGAATT-3′); shTOPBP1#2 (5′-UUCUGAAUCAUGUAAAGGCTT-3′).

These shRNAs were ligated into the pLVX-puro vector to generate shTOPBP1 RNA. The constructs were confirmed by sequencing, and empty vectors served as negative controls. The viral vector was then co-transfected into HEK293T cells, and the viral supernatant was collected. Patu8988, BXPC3, and PANC-1 cells stably expressing shRNA #1 or #2 targeting TOPBP1 were generated after puromycin selection and confirmed by western blotting.

### Cell viability assays

The cell lines (Patu8988, BXPC3, PANC-1, sh-control, and sh-TOPBP1 cells) or primary cells (0001, 0037, 0045) were seeded at a density of 1500 or 5000 cells/well in 96-well plates and incubated for 24 h. And cells were treated with olaparib or AZD6738 at concentrations ranging from 0 to 150 μmol/L or 0 to 40 μmol/L for 72 h. All inhibitor assays were performed in triplicate. Cell proliferation assays were performed using the Cell Counting Kit-8 (CCK8, Dojindo, Japan). Viable cells were quantified at 24 h intervals at 450 nm (OD450) using a microplate reader (Epoch; BioTek, Winooski, VT). IC50 values were determined using a nonlinear regression model by GraphPad Prism software (GraphPad Software 9.5).

### RT-qPCR

Total RNA was extracted from the samples using the RNeasy Plus Kits (Qiagen) according to the manufacturer's instructions. Subsequently, cDNA synthesis was performed using the SuperScript IV Reverse Transcriptase (Invitrogen) following the recommended protocol. Quantitative real-time PCR (RT-qPCR) was conducted using Brilliant III Ultra-Fast SYBR Green QPCR Master Mix (Agilent Technologies) to measure the expression levels of TOPBP1 and GAPDH. Gene-specific primers were designed for amplification. The expression values were normalized to the internal control, GAPDH, and analyzed using the 2^−ΔΔCt^ method.

### Western blot

Cell lysis was achieved using RIPA lysis buffer (Thermo Fisher Scientific), supplemented with Protease Inhibitor Cocktail (MedChemExpress) and Phosphatase Inhibitor Cocktail (Cell Signaling Technology). Western blotting was conducted following standard protocols. The primary antibodies used for western blotting included TOPBP1 antibody (D8G4L, Cat. No. 14342, Cell Signaling Technology) and the DNA Damage Antibody Sampler Kit (Cat. No. 9947, Cell Signaling Technology), which consisted of the following antibodies: p-ATR (Ser428, Cat. No. 2853), p-ATM (Ser1981, Cat. No. 5883), p-BRCA1 (Ser1524, Cat. No. 9009), p-CHK2 (Thr68, Cat. No. 2197), p-CHK1 (Ser345, Cat. No. 2348), γ-H2A.X (Ser139, Cat. No. 9718), and p-P53 (Ser15, Cat. No. 9286). The primary antibodies were purchased from the indicated vendors. Secondary HRP-conjugated antibodies were obtained from Cell Signaling Technology. Detection was performed usingECL-Plus substrate (Bio-Rad) using Bio-Rad ChemiDoc MP imager.

### Human studies

The clinical specimens from patients diagnosed as PDAC at Ruijin Hospital having complete pathologic and clinical information between 2014 and 2023 were collected. The studies involving human subjects were approved by the Ethics Committee of Shanghai Jiaotong University School of Medicine. Tumor samples were diagnosed by the Department of Pathology using hematoxylin and eosin (HE) and immunohistochemistry (IHC) staining techniques.

### Immunohistochemistry (IHC)

IHC staining was performed following a previously described protocol [35]. The primary antibodies used in IHC were anti-TOPBP1 (D8G4L, Cat. No. 14342, CST, 1:800) and anti-Ki67 (Cat. No. 27309, Proteintech, 1:800) diluted in 0.1 M phosphate-buffered saline (PBS). The IHC score was determined by combining the percentage of positively stained cells with the staining intensity score (0, negative or weak; 2, moderate; 3, strong). The score ranged from 0 to 2, representing different percentages of positive cells: 0 (0–20% stained cells), 1 (30–60% positive), and 2 (60–100% positive). The final IHC scores theoretically ranged from 0 to 6, where scores ≥ 3 were considered high and scores < 3 were considered low.

### Bioinformatics analysis

The analysis of gene expression changes was conducted using diverse datasets. The gene expression data of the Patu8988, BXPC3, PANC-1 cell lines were obtained from our RNA-sequencing (RNA-seq) data. The RNA-seq raw counts were normalized, and differential expression analyses were conducted using the DESeq2 R package. Additional datasets were obtained from the Cancer Genome Atlas (TCGA) database and the Gene Expression Omnibus (GEO) website. The TCGA RNaseq datasets were log2 transformed and median-centered, and the GEO datasets (GSE131027 and GSE62452 data) were log-transformed using the mean of probes per gene.

Differential gene expression analysis was performed using DESeq2 and edgeR. To further explore the functional implications of differentially expressed genes, GO and KEGG enrichment analyses were conducted using ClusterProfiler. Additionally, Gene Set Enrichment Analysis (GSEA) was performed using MSigDB hallmark gene sets (H) and curated gene sets (C2). The visualization of differential gene expression and pathways was achieved through the utilization of the ComplexHeatmap package.

### Subcutaneous xenograft model

Pancreatic cancer cell lines were subcutaneously injected nude mice (5 to 6 weeks old) [36]. Each mouse received a 150 μL injection of either Patu8988 WT or shTOPBP cell suspension (1 × 10^6^ cells) into the left flank. Animal health and tumor growth were monitored twice weekly. On day 10 post-implantation, WT and shTOPBP1 mice were randomly assigned to the control (n = 4) or olaparib treatment (n = 4) groups. Olaparib was diluted to 5 mg/mL in 4% DMSO + 30% PEG 300 + ddH_2_O; 50 mg/kg was administered via oral gavage once every 2 days for 20 days. Body weight was evaluated throughout the experiment. The mice were euthanized at 24 h after the final treatment using an intravenous overdose of pentobarbital. All tumors were collected, weighed, and measured using the formula (L × W × W) × (π/6), where L represents the major tumor axis and W represents the minor tumor axis.

### Statistical analysis

The Kaplan–Meier method was used to calculate survival curves. Multiple group analyses were performed using two-way or one-way ANOVA together. The rest experiments were assessed using an unpaired t-test, and statistical significance was set at p < 0.05, considered significant Values were presented as the mean ± standard deviation (SD) (GraphPad Prism 6 program).

## Results

### TOPBP1 expression is upregulated in patients with PDAC and associated with higher histologic grade and shorter survival duration

**Fig. 1:**
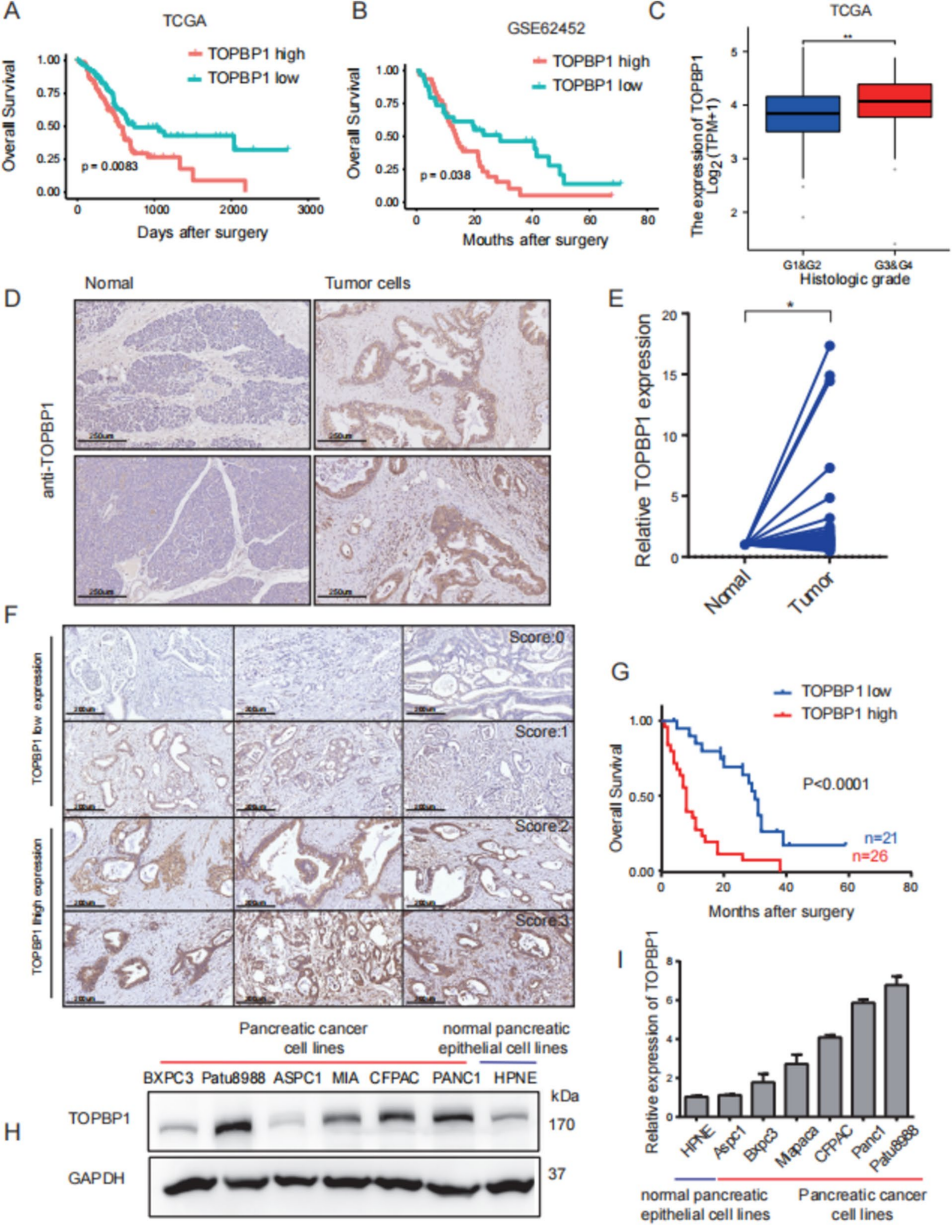
*TOPBP1* expression is upregulated in patients with PDAC and associated with higher histological grade and shorter survival durations. **A** Patients with high *TOPBP1* expression levels have worse overall survival (OS) than those with low *TOPBP1* expression levels (n = 172, p = 0.0083) based on datasets from The Cancer Genome Atlas (TCGA). P-values were derived from the Kaplan–Meier survival analysis. **B** Patients with high *TOPBP1* expression levels have worse OS than those with low *TOPBP1* expression levels based on the Gene Expression Omnibus (GEO) datasets (n = 65, p = 0.038). P-values were derived from the Kaplan–Meier survival analysis. **C** *TOPBP1* expression is higher in PDAC tissues with G3–G4 pathological stage compared to G1–G2 pathological stage in TCGA datasets (n = 179, paired *t*-test, p = 0.003). **D** Immunohistochemistry (IHC) showing *TOPBP1* expression predominantly localized to pancreatic ductal tumor cells within the tumor. **E** Quantitative real-time PCR analysis of *TOPBP1* expression in 58 pairs of PDAC tissues (tumor) compared to normal tissues (normal). **F** *TOPBP1* expression levels in PDAC tissues based on IHC scores. **G** Patients with high *TOPBP1* expression levels had a worse prognosis (OS) than those with low *TOPBP1* levels (n = 47, Kaplan–Meier survival analysis, p < 0.0001) based on our center's patient data. **H** *TOPBP1* expression in various PDAC cell lines analyzed by western blotting. **I** *TOPBP1* expression in various PDAC cell lines analyzed by western blotting. Data shown are the mean ± SEM. Statistical analyses were performed with one-way ANOVA (*p < 0.05 and **p < 0.01 versus control)

To assess the diagnostic and prognostic potential of differential *TOPBP1* expression in PDAC, we analyzed expression data from TCGA and GEO datasets, as well as the survival and clinical data of identified patients. Clinical parameters for 179 patients with PDAC from TCGA were collected and analyzed (Table 1). Elevated *TOPBP1* expression showed a significant correlation with poor overall survival (p = 0.0083 and p = 0.038 for TCGA and GEO datasets, respectively; Fig. 1A, B), advanced pathological stage (Fig. 1C, p = 0.0073), and primary therapy outcome (Fig. S1C, p = 0.0097). However, no significant associations were observed between *TOPBP1* expression and TNM stage, tumor location, degree of nerve and vascular invasion, or distant metastasis (data not shown).

**Table 1:** Association between TOPBP1 expression and clinicopathological features of patients with PDAC using the TCGA database

Characteristics	Variable	Total	TOPBP1		p value
		(n = 179)	Low expression	High expression	
Age (years)	< 65	94 (52.5%)	47 (26.3%)	47 (26.3%)	0.937
	≥ 65	85 (47.5%)	42 (23.5%)	43 (24.0%)	
Sex	Female	80 (44.7%)	40 (22.3%)	40 (22.3%)	0.946
	Male	99 (55.3%)	49 (27.4%)	50 (27.9%)	
Pathologic stage	Stage I	21 (11.7%)	11 (6.2%)	10 (5.7%)	0.674
	Stage II	147 (82.1%)	70 (39.8%)	77 (43.8%)	
	Stage III & IV	8 (4.5%)	5 (2.8%)	3 (1.7%)	
Histologic grade	G1 & G2	127 (70.9%)	72 (40.7%)	55 (31.1%)	0.003^*^
	G3 & G4	50 (27.9%)	16 (9.0%)	34 (19.2%)	
Primary therapy outcome	PD	50 (27.9%)	15 (10.7%)	35 (25.0%)	0.016^*^
	SD & PR & CR	90 (50.3%)	46 (32.9%)	44 (31.4%)	
OS event	Alive	86 (48.0%)	53 (29.6%)	33 (18.4%)	0.002^*^
	Dead	93 (52.0%)	36 (20.1%)	57 (31.8%)	

TCGA: The Cancer Genome Atlas

^*^p < 0.05 was considered to denote statistical significance

To investigate and validate the role of TOPBP1 in PDAC tumorigenesis, we analyzed PDAC patient samples from our center. IHC and quantitative real-time PCR (qPCR) assays were performed, revealing predominant localization of TOPBP1 within the pancreatic ductal tumor cells (Fig. 1D, E). By scoring TOPBP1 expression through IHC and combining it with clinical data, we observed a significant association between high TOPBP1 expression and poor overall survival (Fig. 1F, G, Table 2). Additionally, we evaluated the baseline *TOPBP1* expression levels in PDAC cell lines, finding higher levels in pancreatic cancer cell lines than in normal pancreatic cell lines (Fig. 1H, I).

**Table 2:** Correlation between TOPBP1 expression and clinicopathological features of patients with PDAC (n = 47)

Characteristics	Variable	Total	TOPBP1		p value
		(n = 47)	Low expression	High expression	
Age (years)	< 60	16 (34%)	6 (37.5%)	10 (62.5%)	0.547
	≥ 60	31 (66%)	15 (48.4%)	16 (51.6%)	
Sex	Female	17 (36.2%)	12 (70.6%)	5 (29.4%)	0.014^*^
	Male	30 (63.8%)	9 (30%)	21 (70%)	
Differentiation	Poorly	9 (19.2%)	1 (11.1%)	8 (88.9%)	0.03
	Moderately or well	38 (80.9%)	20 (52.6%)	18 (47.4%)	
Tumor size	< 3	8 (17%)	7 (87.5%)	1 (12.5%)	0.015^*^
	≥ 3	39 (83%)	14 (35.9%)	25 (64.1%)	
Nerve invasion	No	8 (17%)	5 (62.5%)	3 (37.5%)	0.437
	Yes	39 (83%)	16 (41%)	23 (59%)	
Vascular invasion	No	35 (74.5%)	21 (60%)	14 (40%)	0.38
	Yes	14 (29.8%)	2 (14.3%)	12 (85.7%)	
Lymph node metastasis	No	20 (42.6%)	9 (45%)	11 (55%)	1
	Yes	27 (57.5%)	12 (44.4%)	15 (55.6%)	
TNM stage	I + II	14 (29.8%)	8 (57.1%)	6 (42.9%)	0.342
	III + IV	33 (70.2%)	13 (39.4%)	20 (60.6%)	
Tumor location	Head & neck	26 (55.3%)	12 (46.2%)	14 (53.9%)	1
	Body & tail	21 (44.7%)	9 (42.9%)	12 (57.1%)	
Distant metastasis	No	40 (85.1%)	20 (50%)	20 (50%)	0.112
	Yes	7 (14.9%)	1 (14.3%)	6 (85.7%)	

^*^p < 0.05 was considered to denote statistical significance

Taken together, our findings implicate TOPBP1 in PDAC progression and holds potential as a predictive biomarker for the treatment of patients.

### Correlation between TOPBP1 and the DDR pathway in PDAC

**Fig. 2:**
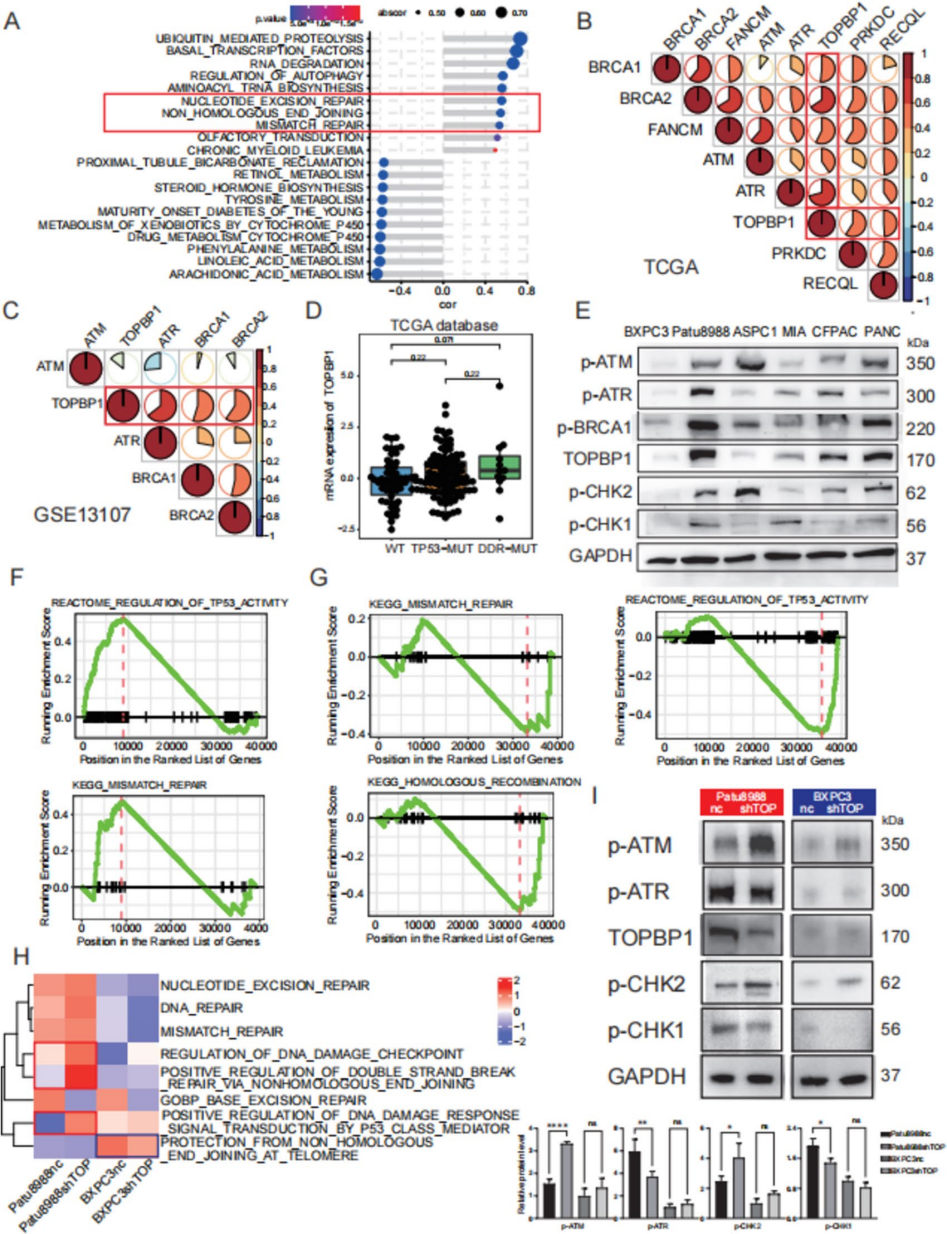
Expression levels of TOPBP1 correlate with changes in the DDR pathway and DNA replication. **A** Correlation analysis between *TOPBP1* expression levels and KEGG signaling pathways. **B** Correlation analysis between *TOPBP1* expression levels and key DDR pathway-related genes using datasets from The Cancer Genome Atlas (TCGA). **C** Correlation analysis between *TOPBP1* expression levels and key DDR pathway-related genes using Gene Expression Omnibus (GEO) datasets. **D** Differential expression levels of *TOPBP1* in pancreatic ductal adenocarcinoma (PDAC) patients classified as wild-type (WT), DDR pathway gene-mutated, and *TP53*-mutated. **E** Expression levels of *TOPBP1* and other key DDR-related genes in various PDAC cell lines determined by western blot. **F**, **G** Representative DDR signaling pathways shown by gene set enrichment analysis (GSEA) in Patu8988 and BXPC3 cells after *TOPBP1* knockdown. **H**. Gene ontology (GO) biological process (BP) analysis of differences in signaling pathway enrichment in Patu8988 cells after *TOPBP1* knockdown. **I** Differential changes in DDR genes analyzed by western blot and ImageJ in the PDAC cell lines Patu8988 and BXPC3 after *TOPBP1* knockdown

The STRING Interaction Network revealed a close interaction relationship between TOPBP1 and key regulators of the DDR pathway, including ATR and ATM (Fig. S1D). To assess the correlation between TOPBP1 and DDR pathways in PDAC, we extracted expression and mutation data from TCGA and GEO datasets. Our analysis of TCGA datasets showed a close correlation between *TOPBP1* mRNA expression and various DDR-associated pathways, including NER, NHEJ, and mismatch repair (Fig. 2A). We also observed a correlation between *TOPBP1* mRNA expression and key DDR pathway-associated genes (Fig. 2B, C) in TCGA and GEO datasets. Moreover, *TOPBP1* mRNA expression was upregulated in the presence of DDR pathway related mutations (Fig. 2D); co-occurrence of mutations in *TOPBP1* and key DDR pathway genes was frequent in PDAC (Fig. S2E, F).

In PDAC cancer cell lines, we evaluated the abundances of TOPBP1, p-ATR, p-ATM, p-CHK1, and p-CHK2 proteins—key genes in the ATR and ATM pathways. These pathways play an important role as upstream sensing pathways for the DDR pathway. And we observed a positive correlation between changes in protein abundance of TOPBP1 and p-ATR, and p-CHK1, in PDAC cell lines. These correlations exhibited consistent trends in expression levels (Fig. 2E). We selected Patu8988 and BXPC3 as the TOPBP1-high and TOPBP1-low cells lines, respectively, for further investigation.

Taken together, these results confirm the strong association between TOPBP1 and the DDR pathway, as well as the ATR and ATM pathways, which act upstream of the DDR pathway.

### *TOPBP1* knockdown inhibits the ATR pathway, increases burden on the ATM pathway, and elicits distinct DDR responses

To further clarify the role of TOPBP1 in the DDR pathway, we generated stable *TOPBP1*-knockdown cells by infecting them with lentiviral shRNAs. The success of the *TOPBP1* knockdown was confirmed by western blot analysis (Fig. S2A). Initially, we investigated the impact of *TOPBP1* knockdown on cell cycle and proliferation in *TOPBP1*-knockdown Patu8988 cell lines. RNA sequencing revealed altered pathways, including negative regulation of cell cycle processes, spindle organization (Fig. S2B). However, in vitro experiments showed that *TOPBP1* knockdown alone resulted in minor changes in cell proliferation (Fig. S2C) and apoptosis (Fig. S2D), although these changes were not statistically significant.

Based on the RNA sequencing results for *TOPBP1*-knockdown cell lines, we observed significant variability in DDR changes between the Patu8988 and BXPC3 cell lines. Patu8988, characterized by high *TOPBP1* expression, exhibited upregulation in pathways related to TP53 activity and mismatch repair (Fig. 2F). In contrast, BXPC3, with low *TOPBP1* expression, showed downregulation of pathways associated with TP53 activity, mismatch repair, and homologous recombination (Fig. 2G). And other DDR signaling pathways, namely base excision repair and non-homologous end joining, exhibited similar changing trends in both Patu8988 and BXPC3 cells following *TOPBP1* knockdown (Fig. S3A, B). To provide a more intuitive visual representation of these pathway changes, we conducted GO analysis and generated heat maps (Fig. 2H). At the protein level, knockdown of TOPBP1 resulted in a moderate decrease in p-CHK1 levels and a marked increase in p-ATM and p-CHK2 levels (Fig. 2I). This effect was particularly pronounced in Patu8988 cells. Based on these observations, we hypothesize that TOPBP1 knockdown impairs the ATR pathway while concurrently increasing the burden on the ATM pathway.

These findings suggest that varying levels of TOPBP1 may reflect different burdens on the ATM and ATR pathways, potentially influencing diverse responses within the DDR.

### *TOPBP1* may be a critical point for *BRCA1* non-mutated and PARP-sensitive groups by increasing the load on the ATM pathway

**Fig. 3:**
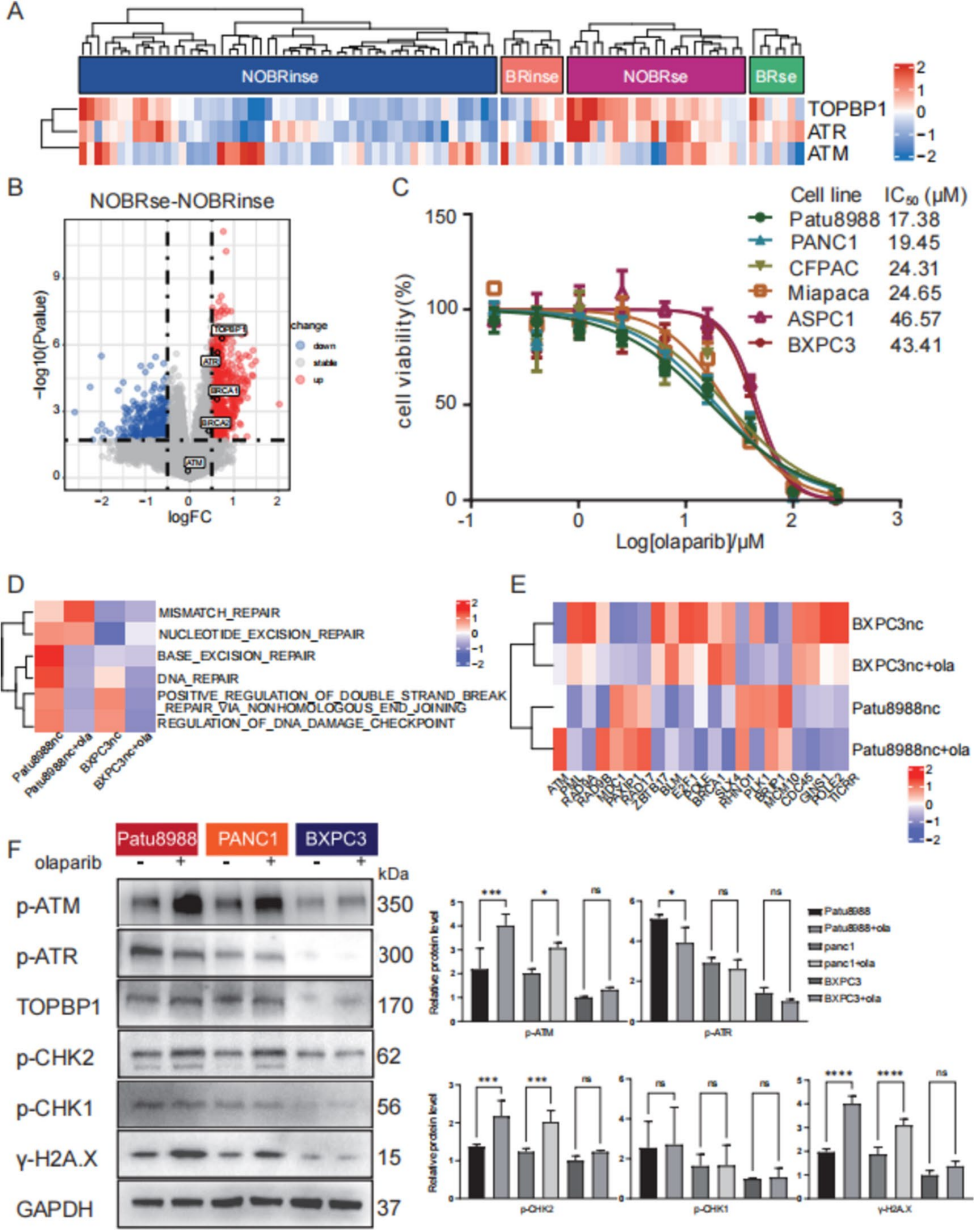
The influential role of TOPBP1, ATR, ATM, and DDR pathways in the inhibitory effect of olaparib on pancreatic cell proliferation. **A** Heat map of the differential TOPBP1, ATR, and ATM gene expression in four groups based on *BRCA1* mutation status and PARP sensitivity: NOBRinse (*BRCA1* non-mutation and PARP insensitivity), BRinse (*BRCA1* mutation and PARP insensitivity), NOBRse (*BRCA1* non-mutation and PARP sensitivity), and BRse (*BRCA1* mutation and PARP sensitivity). **B** Volcano plots illustrating the increased expression of *TOPBP1* and *ATR* in comparison to the *BRCA1* non-mutation and PARP-sensitivity groups, as well as the *BRCA1* non-mutation and PARP-insensitivity groups. **C** Determination of PDAC cell line sensitivity to olaparib using the CCK8 assay. **D** Heatmap showing differences in signaling pathway enrichment alterations induced by olaparib among BXPC3 and Patu8988 cells treated with olaparib based on Gene Ontology (GO) Biological Processes (BP). **E** Heatmap showing differential gene expression in BXPC3 and Patu8988 cells under olaparib treatment. **F** Differential changes in *TOPBP1* and other DDR genes analyzed by western blot and ImageJ in PDAC cell lines treated with olaparib and expressing different levels of *TOPBP1*

PARPi-resistant *BRCA1*-deficient cells are more reliant on ATR for survival than BRCA1-proficient cells [27, 29]. The combination of DNA-damaging drugs, including PARP inhibitors and alternative DDR inhibitors, is introducing a new era in PDAC therapy, aiming to exploit artificial vulnerabilities termed "HRDness inducers" [27, 29]. In our analysis of the GEO database, including tumors with *BRCA1* mutations and PARP sensitivity data, we categorized the samples into four groups: *BRCA1* non-mutation and PARP insensitivity, *BRCA1* mutation and PARP insensitivity, *BRCA1* non-mutation, and PARP sensitivity, and *BRCA1* mutation and PARP sensitivity. Focusing on the *BRCA1* non-mutation and PARP sensitivity group, we found unique enrichment in specific pathways, such as DNA replication, single-stranded DNA binding, and damaged DNA binding (Fig. S3C, D). Moreover, *TOPBP1* and *ATR* were significantly overexpressed in this group (Fig. 3A, B).

We assessed the drug sensitivity of pancreatic ductal adenocarcinoma (PDAC) cell lines to olaparib, a potent PARP inhibitor. Our findings revealed that cells with high TOPBP1 expression, such as Patu8988 and PANC1, exhibited greater sensitivity to olaparib compared to cells with low TOPBP1 expression, such as ASPC1 and BXPC3 (Fig. 3C). Although the observed differences in sensitivity were not strikingly large, they suggest a potential correlation between TOPBP1 expression levels and the response to olaparib treatment.

Following treatment with a low-dose (5 μM) of olaparib, we investigated changes in the DDR pathway via RNA sequencing and western blot analyses. PDAC cells with varying levels of TOPBP1 expression, namely Patu8988 and BXPC3, exhibited similar trends in expression changes associated with the DDR pathway but displayed widely differing degrees of variability (Fig. 3D). Furthermore, genes associated with *TOPBP1* demonstrated different responses, with *ATM* being significantly upregulated specifically in Patu8988 cells (Fig. 3E).

We also explored changes in the ATM and ATR pathways via western blotting. The results revealed upregulation of p-ATM and p-CHK2 following low-dose olaparib treatment in PDAC cells with high TOPBP1 expression (Patu8988), while cells with low TOPBP1 expression (BXPC3) exhibited less pronounced changes (Fig. 3F). These findings suggest that olaparib can enhance ATM pathway activation in PDAC cells exhibiting high TOPBP1 expression, leading to an intensified burden on this pathway. Hence, pancreatic tumor cells expressing high levels of *TOPBP1* may exhibit heightened sensitivity to olaparib, leading to an exacerbation of the ATM pathway burden.

### *TOPBP1* downregulation enhances the inhibitory effect of olaparib on pancreatic cell proliferation

**Fig. 4:**
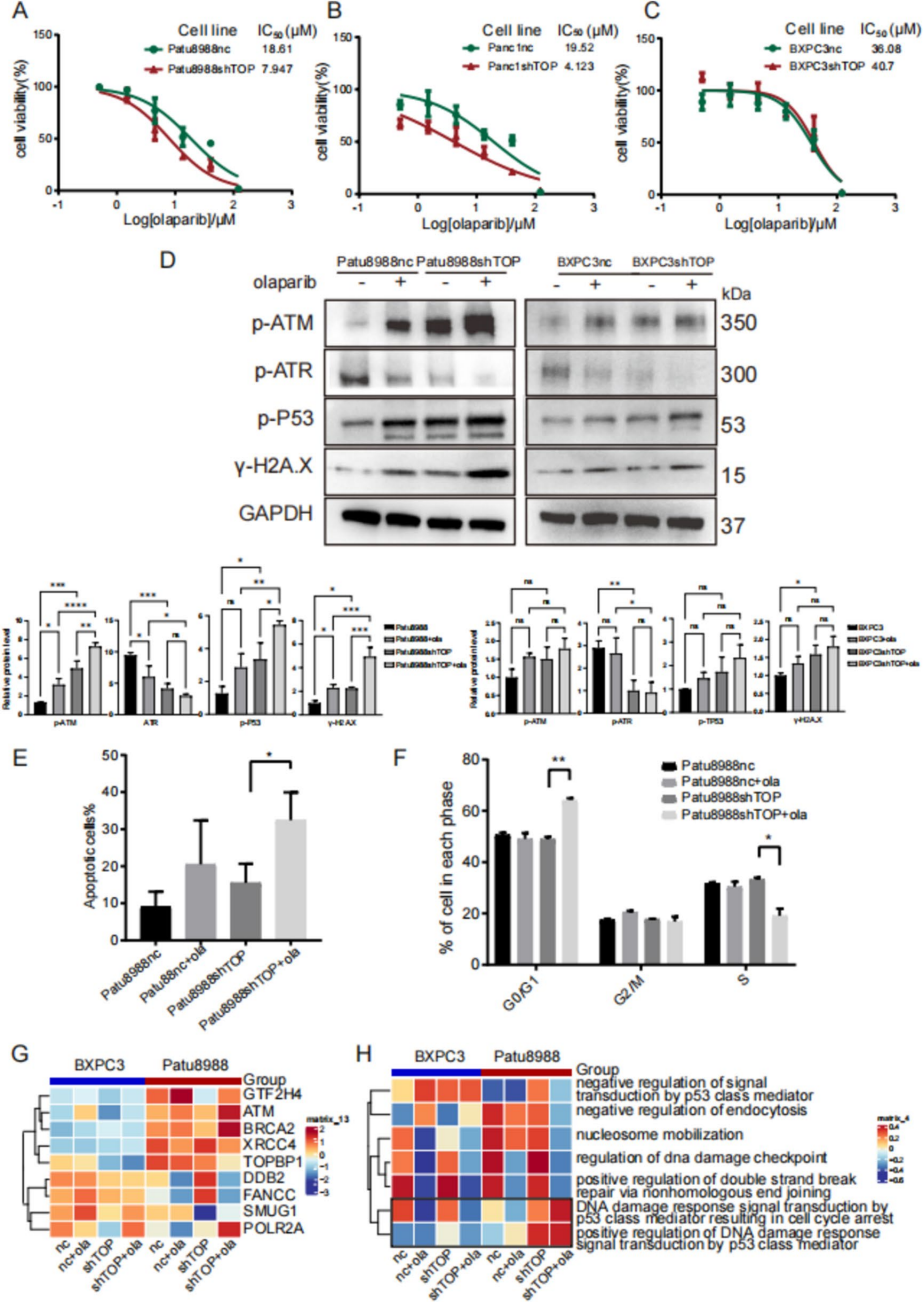
Pathway changes in PDAC cell lines with different *TOPBP1* levels after olaparib and *TOPBP1*-knockdown treatment. **A**–**C** IC_50_ values determined by the CCK8 assay showing that *TOPBP1* knockdown impairs the drug sensitivity of Patu8988 and Panc1 cells to olaparib compared to the control treatment; this is not apparent in BXPC3 cells. **D** Differential changes in DDR genes analyzed by western blot and ImageJ in Patu8988 and BXPC3 cells after *TOPBP1* knockdown and treatment with 5 μM olaparib. **E**. Effect of olaparib on apoptosis in Patu8988 nc or shTOPBP1 cells assessed by FACS; the proportion of Annevin V/7-AAD cells is shown. PDAC cells were treated with 5 μM olaparib for 48 h. Significant differences were observed between *TOPBP1*-knockdown and control cells after olaparib treatment. **F** Effect of olaparib on cell cycle arrest analyzed by FACS; the proportion of cells positive for FL2-H-PI staining is shown. Patu8988 WT or *TOPBP1*-knockdown cells were treated with 5 μM olaparib for 48 h; combined olaparib and *TOPBP1* knockdown arrested cells in the G0/G1 phase. **G** Heatmap showing differential gene expression in BXPC3 and Patu8988 cells under control conditions, olaparib treatment, *TOPBP1* knockdown, or both. **H** Heatmap of gene set enrichment analysis (GSEA) used to analyze differences in KEGG signaling pathway enrichment among BXPC3 and Patu8988 cells treated with olaparib, *TOPBP1* knockdown, or both. The P53 signaling pathway was upregulated in the *TOPBP1*-knockdown Patu8988 cells treated with olaparib, but not in BXPC3 cells

The impact of olaparib on cell proliferation was assessed in PDAC cells following TOPBP1 inhibition. Three commercially available *TOPBP1*-knockdown pancreatic cancer cell lines, namely Patu8988, PANC1, and BXPC3, were treated with olaparib (Fig. 4A, C). In *TOPBP1*-knockdown cells, the IC_50_ values of olaparib ranged from 18.6 μM to 7.95 μM in Patu8988 cells, 19.5 μM to 4.12 μM in PANC1 cells, and 36.08 μM to 40.7 μM in BXPC3 cells.

Subsequently, changes in key factors, including p-ATR, p-ATM, p-P53, and γ-H2A.X, in the DDR pathway of PDAC cells subjected to *TOPBP1* knockdown and treated with 5 μM olaparib were analyzed via western blotting (Fig. 4D). Compared to Patu8988nc cells, the knockdown of TOPBP1 in Patu8988 cells resulted in a decrease in p-ATR, indicating a loss of ATR pathway activity. This was accompanied by a slight increase in ATM load, as well as significant upregulation of p-P53 and γ-H2A.X. Upon treatment with olaparib, inactivation of the ATR pathway further led to overloading and unsustainability of the ATM pathway, which in turn caused enhanced activation of p-P53 and γ-H2A.X. In contrast, these effects were not observed in BXPC3 cells, which exhibit low TOPBP1 expression.

Furthermore, we assessed changes in cell cycling and apoptosis in *TOPBP1*-knockdown Patu8988 cells under treatment with olaparib or control conditions. *TOPBP1* knockdown, when combined with olaparib treatment, resulted in significant apoptosis (Fig. 4E) and induced cell cycle arrest in the G0/G1 phase, along with a reduction in cells in the S phase (Fig. 4F). These findings indicate that the combined effect of these two treatments increases the ATM load, resulting in cell cycle arrest and cell death.

### Differential regulation of signature pathways in cell lines with varying *TOPBP1* levels upon olaparib treatment

We then investigated the genes and pathways associated with responses to olaparib treatment and *TOPBP1* knockdown on DNA damage and PDAC tumor cell proliferation through RNA sequencing. Results revealed a substantial increase in *ATM, BRCA2*, and *POLR2A* expression upon *TOPBP1* knockdown and treatment with olaparib in Patu8988 cells, which was consistent with the observed protein expression level changes (Fig. 4G). KEGG, Reactome enrichment analysis indicated that the different levels of *TOPBP1* knockdown and olaparib treatment resulted in diverse pathway outcomes (Fig. S3E, F).

Olaparib treatment slightly enhanced DDR signal transduction through the P53 mediator in *TOPBP1*-knockdown Patu8988 cells (Fig. 4H), whereas these effects were not observed in BXPC3 cells. Additionally, we co-immunoprecipitated TOPBP1 and TP53 and found that they bound together in Patu8988 and BXPC3 cells (Fig. S3G).

Based on these observations, we hypothesized that reducing *TOPBP1* expression could trigger P53 release and enhance the ATM pathway burden, promoting activation of TP53-mediated cell death and inhibition of tumor growth, particularly in cells with high *TOPBP1* expression levels. Inhibition of the TOPBP1-regulated ATR pathway could play a critical role in improving olaparib responses in cell lines with high *TOPBP1* expression. This inhibition would lead to an overload of the ATM pathway and subsequent TP53-induced cell death.

### Downregulation of TOPBP1 sensitizes animal models to olaparib

**Fig. 5:**
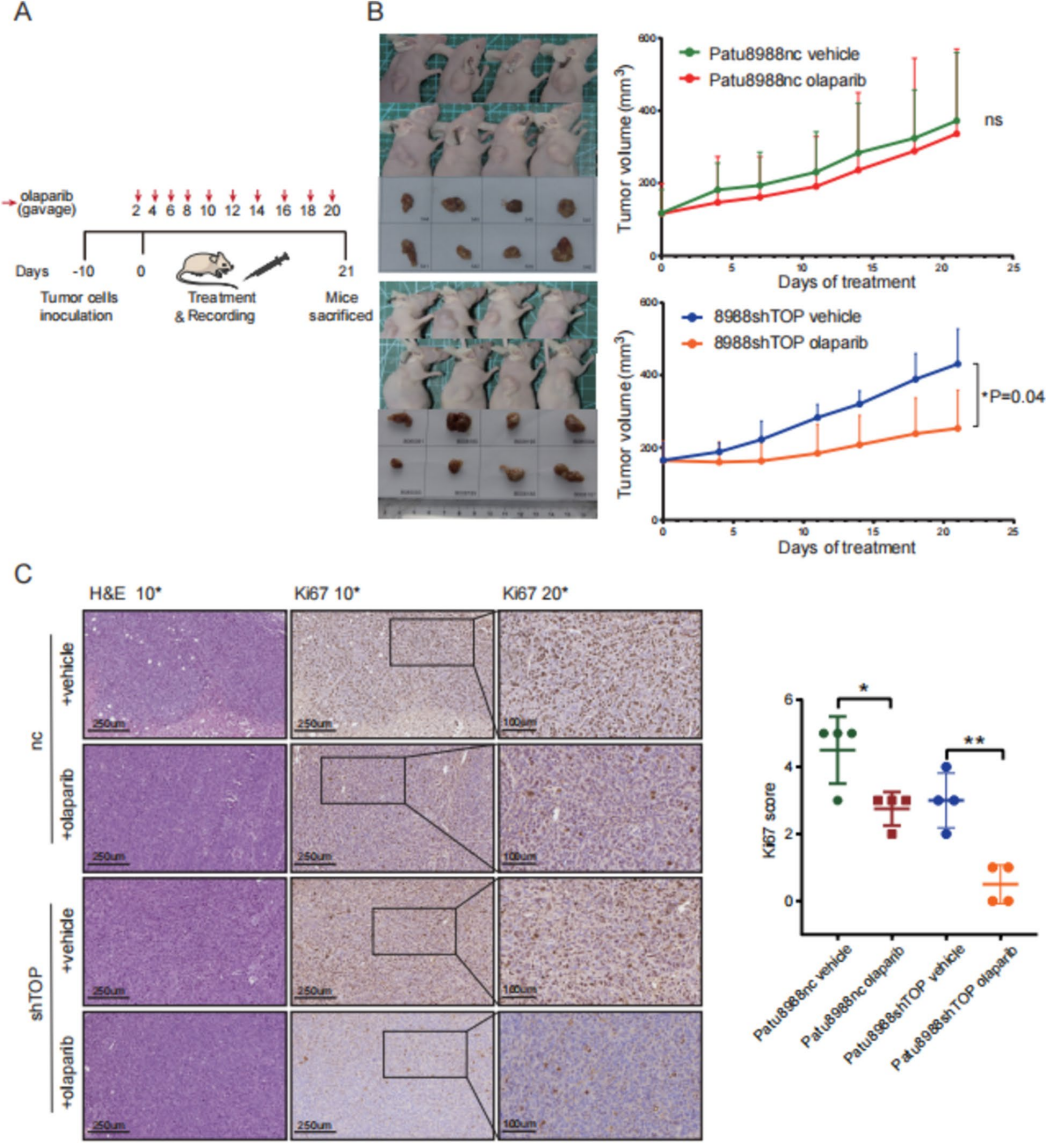
Downregulation of TOPBP1 sensitizes animal models to olaparib. **A** Schematic diagram of the mouse model. **B** Tumor volumes were measured in implanted mouse models of Patu8988 nc and *Topbp1*-knockdown following 21 days of olaparib treatment. Results show that *Topbp1* knockdown increased drug sensitivity to olaparib in mice injected with Patu8988 cells. **C** Hematoxylin and eosin (HE) and Ki67 immunochemical staining were performed on tumor tissues from Patu8988 nc and *Topbp1*-knockdown mice treated with either vehicle or olaparib. The positive Ki67 staining was significantly reduced in *Topbp1*-knockdown tumors treated with olaparib compared to WT tumors treated with olaparib

The efficacy of olaparib after *Topbp1* knockdown was further evaluated in a xenograft mouse model of PDAC using subcutaneously implanted Patu8988 WT and shTOPBP1 cells. Nude mice were implanted with PDAC cells in the right axilla, and ten days later, they were randomly assigned to treatment groups. The treatment groups received either olaparib (administered via gavage) or a vehicle control (administered intraperitoneally) at specific time intervals (Fig. 5A). On day 21 post-treatment, tumors were harvested. A significant reduction in tumor volumes was observed in the olaparib-treated mice injected with *Topbp1*-knockdown Patu8988 cells compared to those in the vehicle group (Fig. 5B, Fig. S4A, B). Notably, all groups showed an acceptable level of weight loss (Fig. S4C, D). In contrast, no significant change in tumor volume was observed in olaparib-treated mice injected with Patu8988 WT cells (Fig. 5B and Fig. S4A, B).

Moreover, the Ki67-based mitotic proliferation index demonstrated that olaparib effectively inhibited tumor growth in mice injected with *Topbp1*-knockdown Patu8988 cells (Fig. 5C). Following *Topbp1* knockdown, we hypothesized that olaparib effectively impedes cancer growth in an orthotopic model utilizing the Patu8988 cell line.

Collectively, these results demonstrated that in a subcutaneously implanted xenograft mouse model, olaparib effectively suppressed the growth of pancreatic cancer cells following *Topbp1* knockdown in Patu8988 cells characterized by high *Topbp1* expression.

### TOPBP1 as a predictor of PDAC response to combination therapy involving olaparib and AZD6738

Targeting the ATR pathway holds great promise for anticancer therapy, as tumor cells under substantial replication stress rely heavily on ATR for their survival. [37]. The sensitization of TOPBP1-high PDAC cells to olaparib treatment following *TOPBP1* knockdown is primarily attributed to ATR pathway inhibition and ATM exacerbation, leading to P53 release and subsequent activation of TP53-induced cell death. Given the lack of established TOPBP1 inhibitors, AZD6738—a potent ATR kinase inhibitor [38]—has garnered significant interest in cancer research [39, 40]. Hence, we examined the effects of combining olaparib and AZD6738 in cells exhibiting varying levels of *TOPBP1*.

**Fig. 6:**
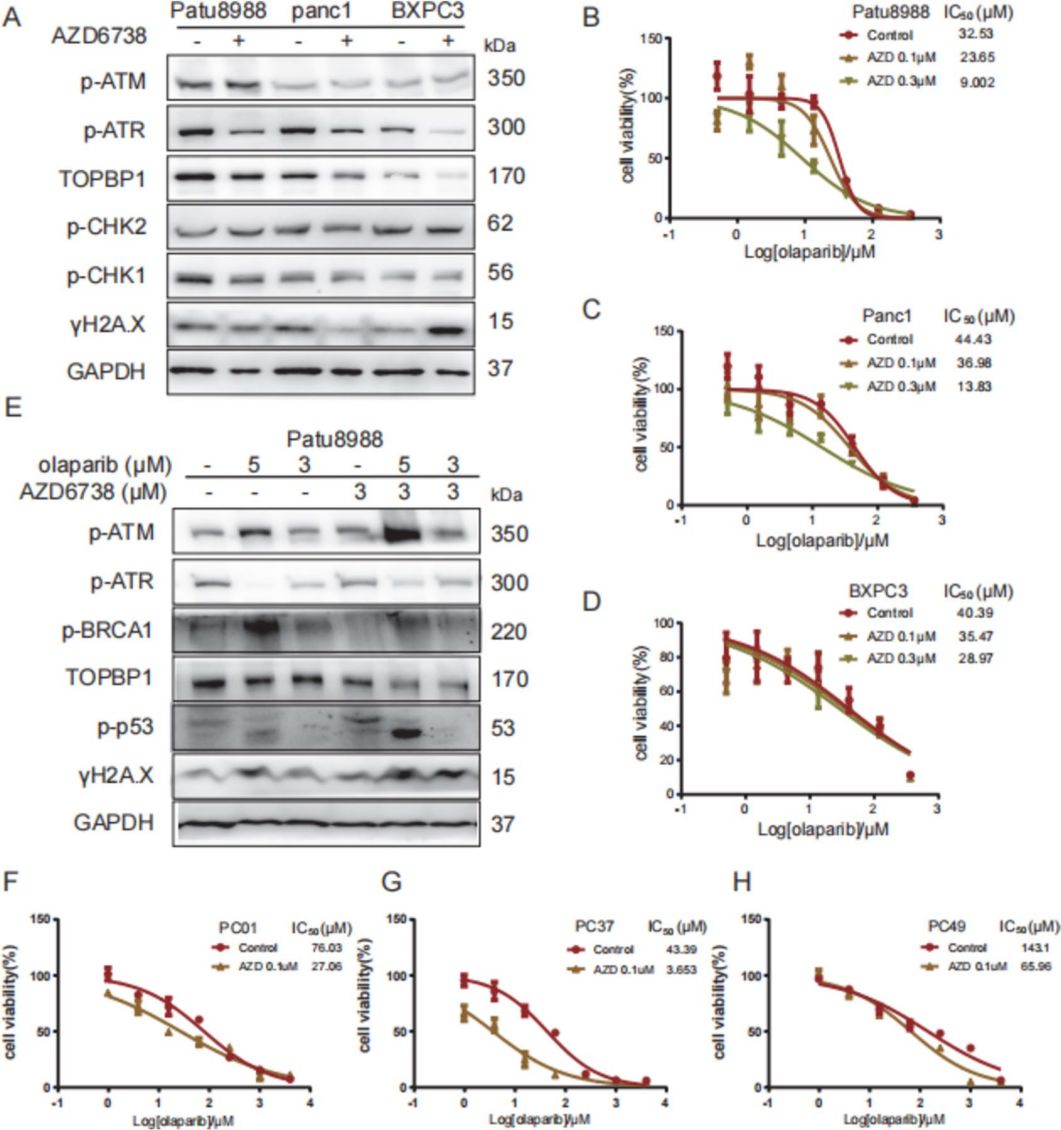
Targeting TOPBP1 as a predictor of the effectiveness of combined olaparib and ATRi drug therapy in PDAC cell lines and PDAC primary cells. **A** Differential changes in TOPBP1 and other proteins encoded by key DDR-related genes in the PDAC cell lines Patu8988, PANC1, and BXPC3 after treatment with AZD6738. **B**–**D** ATRi and AZD6738 impaired the drug sensitivity of Patu8988 and PANC1 to olaparib compared to the control; this effect was not significant in BXPC3 cells. **E** Differential changes in DDR genes in Patu8988 cells treated with 5 μM or 3 μM olaparib alone or in combination with 3 μM AZD6738. **F**–**H** Drug sensitivity changes in primary PDAC cells (0001, 0037, and 0049) to olaparib alone or in combination with AZD6738. IC_50_ values determined by the CCK-8 assay showed that combined treatment with AZD6738 increased drug sensitivity to olaparib, particularly in 0001 and 0037, this effect was not observed in 0049 primary cells

Diminished sensitivity to AZD6738 was detected in PDAC cells exhibiting high *TOPBP1* expression (Fig. S5A). Additionally, low-dose AZD6738 treatment led to reduced levels of TOPBP1, p-ATR, and p-CHK1, confirming the efficacy of ATR inhibition in suppressing the ATR pathway and downregulating *TOPBP1* expression (Fig. 6A).

PDAC cells with high *TOPBP1* expression exhibited increased sensitivity to the combined olaparib and AZD6738 treatment compared to cells with low *TOPBP1* expression (Fig. 6B–D). Western blot analysis further revealed that the combination of 5 μM olaparib and 3 μM AZD6738 suppressed the ATR pathway and exacerbated the ATM pathway burden, leading to elevated p-P53 expression and induction of apoptosis (Fig. 6E).

The effects of the combined treatment with olaparib and AZD6738 on primary PDAC tumor cells (PC01, PC37, and PC49) were assessed. The basal expression levels of TOPBP1 and key factors involved in the DNA damage response (DDR) pathway were evaluated in these primary cells (PC01, PC37, and PC49) by Western blotting (Fig. S5B). Additionally, the sensitivity of these cells to olaparib and the ATR inhibitor (ATRi) was determined (Fig. S5C, D). Notably, PC37 cells, which exhibited higher levels of TOPBP1 expression, demonstrated greater sensitization to the combination of olaparib and AZD6738 compared to PC49 and PC01 cells, which had lower TOPBP1 expression. Olaparib treatment significantly inhibited the proliferation of pancreatic cancer cells, with IC50 values ranging from 43.39 μM to 3.653 μM in PC37 cells, from 76.03 μM to 27.06 μM in PC01 cells, and from 143.1 μM to 65.96 μM in PC49 cells (Fig. 6F, H).

These findings suggest that the baseline level of TOPBP1 in cells may serve as an indicator of sensitivity to combinatorial olaparib and AZD6738 treatment in PDAC cell lines and primary PDAC tumor cells. Therefore, for patients with PDAC and high *TOPBP1* expression, combination therapy comprising olaparib and AZD6738 may offer clinical benefits.

## Discussion

PDAC represents one of the most formidable and aggressive forms of cancer. Regrettably, the efficacy of available targeted therapies for PDAC remain significantly limited [41]. Previous research has demonstrated that pancreatic cancer cells undergo alterations in DDR mechanisms, which contribute to their genomic instability and heightened mutation rates. Furthermore, the DDR pathway plays a critical role in determining the sensitivity and resistance of cancer cells to cytotoxic treatments [18, 42]. In this study, we explored the DDR pathway as a means to identify novel targets and therapeutic strategies for PDAC. Our investigation focused on TOPBP1—a pivotal protein involved in the DDR pathway—with the aim of discovering new targets and therapies for PDAC. We have provided compelling evidence to support TOPBP1 as a novel predictive target that influences the effects of combined olaparib and ATR inhibitor treatment.

We conducted a systematic analysis of the baseline expression levels of TOPBP1, ATR, and ATM pathways in PDAC. Utilizing TCGA data, our clinical samples, and pancreatic cell lines, we confirmed the overexpression of *TOPBP1* in pancreatic cancer cells and tissues. Moreover, we observed a significant association between elevated *TOPBP1* expression and advanced pathological stage as well as poor overall survival. Furthermore, a positive correlation was detected between *TOPBP1* expression and the ATR pathway in the cellular context of PDAC cancer cell lines.

TOPBP1 plays a crucial role in DDR by facilitating the clustering of DNA damage sensors, mediators, and effectors at damaged sites. This allows the formation of complexes with ATR and ATRIP [43, 44]. Moreover, TOPBP1 is recruited to DNA break sites, where it stimulates the kinase activity of ATR [43, 44]. Acting as a scaffolding hub, TOPBP1 assembles multiple protein ligands and directs their binding to intact or fragmented chromatin structures, allocating them to various DNA metabolic pathways [8, 45]. To assess the contribution of TOPBP1 to DDR and ATR pathways, as well as explore its potential as a target for PDAC therapy, we generated stable *TOPBP1*-knockdown cells. As anticipated, the knockdown of *TOPBP1* suppressed the ATR pathway, leading to an increased reliance on the ATM pathway and ultimately accumulation of DNA damage. This suggests that *TOPBP1* knockdown may serve as a potential inducer of homologous recombination deficiency (HRDness).

Previous studies have shown that inhibiting *TOPBP1* expression or activity can sensitize cancer cells to chemotherapy and radiation therapy [15, 46]. This is due to the increased susceptibility of cancer cells to DNA damage and their reduced inability to repair the damage efficiently. Building on the observation that TOPBP1 may be a potential “HRDness inducer,” [13, 47] we used olaparib, typically administered to HR-deficient tumor patients, and narrowed its use to 5–7% of patients with PDAC with a pathogenic germline *BRCA* mutation, aiming to establish a new PDAC treatment strategy. As anticipated, shTOPBP1 sensitized TOPBP1-high PDAC cells, while having no such effect on TOPBP1-low cells, when subjected to olaparib treatment. This distinction suggests that there may be differential regulation of the DDR pathway in PDAC cells with high and low TOPBP1 levels.

High TOPBP1 levels in the presence of ATRi or *TOPBP1* knockdown may induce HRDness and sensitize cells to olaparib treatment. Our findings indicate that cells with elevated *TOPBP1* expression experience significant replication stress, resulting in increased DNA damage accumulation and enhanced sensitivity to PARP inhibition, particularly when ATR is simultaneously suppressed. Simultaneous administration of olaparib and AZD6738 markedly decreased tumor cell viability and imposed a substantial load on ATM, triggering P53-mediated apoptosis in pancreatic cell lines and primary cells.

Our observations further demonstrate the involvement of TP53 in the response of cancer cells to combined olaparib, shTOPBP1, or AZD6738 treatment. ATR and ATM have the capacity to activate P53, which subsequently triggers an apoptotic response. Previous studies have suggested that TOPBP1 may play a critical role in facilitating the gain-of-function activities of mutant P53 oncogenes [48–50], potentially contributing to the development of malignant human cancers in the presence of *P53* mutations. Several studies have demonstrated that P53 variants can interact with TOPBP1, leading to a reduction in the checkpoint response to replication stress while promoting replication during cancer progression [36, 50]^.^ Additionally, we observed that the knockdown of TOPBP1 releases P53 and triggers P53-dependent apoptosis in PDAC cells. However, the intricate relationship between TOPBP1 and P53 was not thoroughly investigated, highlighting the need for further in-depth exploration. Such investigation has the potential to significantly advance the development of P53-targeted drugs.

Combining olaparib with inhibitors downstream of the DDR pathway is gaining increasing interest in solid tumor treatment. Indeed, ATR plays a crucial role in the survival of PARPi-resistant *BRCA1*-deficient cells. Inhibitors of ATR effectively impede the progression of replication forks and disrupt BRCA1-independent RAD51 loading into DNA double-strand breaks, overcoming resistance mechanisms [30, 51]. In pancreatic cancer cells, the combined treatment of olaparib and AZD6738 is associated with a more significant reduction in radiation survival compared to either medication alone, regardless of the patient's HR status [29]. Through co-immunoprecipitation, we observed an interaction between PARP1 and TOPBP1 in pancreatic cancer cell lines, indicating the involvement of complex molecular regulatory mechanisms in the combined effects of olaparib with shTOPBP1 or ATRi. Therefore, these intricate regulatory mechanisms necessitate further investigation.

Numerous targeted therapies are being developed for the treatment of pancreatic cancer, aiming to exploit deficiencies in the DNA damage repair mechanisms. These therapies specifically target proteins involved in the DDR pathway and have exhibited promising results in preclinical studies. However, further clinical trials are necessary to assess their efficacy and safety.

**Fig. 7:**
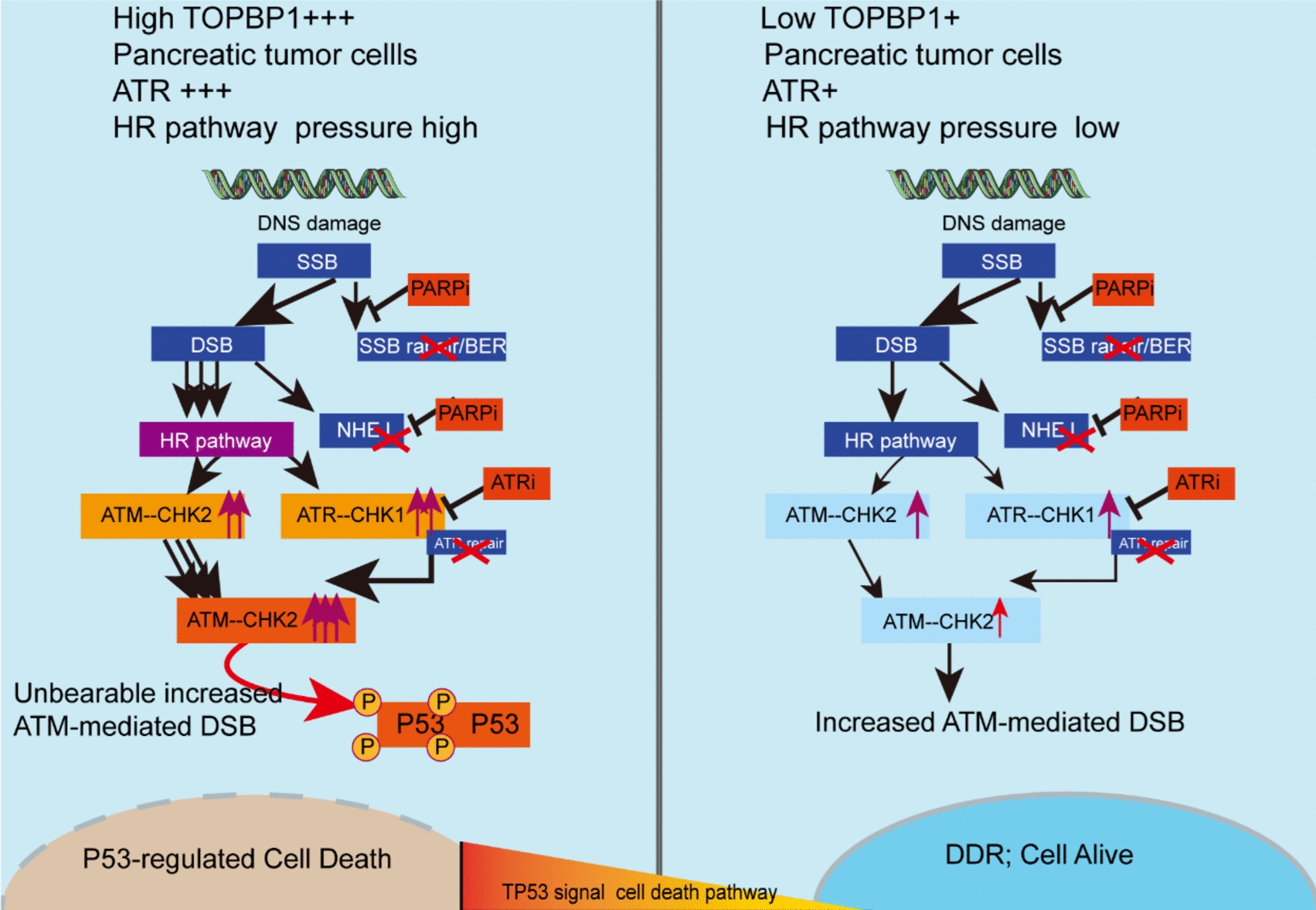
Putative model of the key mechanisms influencing high *TOPBP1* expression in PDAC tumor cells, targeted by combined PARPi (olaparib) and ATRi (AZD6738) therapy

In this study, we comprehensively investigated and elucidated the intricate relationship between TOPBP1, the DDR pathway, and therapeutic responses in PDAC without BRCA1 mutation (Fig. 7). Based on our findings, we propose TOPBP1 as a potential predictive marker that can optimize the application of combination olaparib and AZD6738 therapy for PDAC. We anticipate that prognostication based on TOPBP1 will significantly enhance treatment outcomes and benefit a wider range of patients with PDAC.

## Supplementary Information

Supplementary Material 1. Fig. S1. TOPBP1 is closely associated with the DDR pathway. A–B. TOPBP1 expression in pan-cancer TCGA sequencing data assessed using independent or paired sample. C. TOPBP1 expression was higher in PDAC patients with progressive disease (PD) therapy outcomes compared to those with complete response (CR), stable disease (SD), or partial response (PR) therapy outcomes based on TCGA datasets (n = 179, p = 0.016). D. TOPBP1 gene interaction network based on gene–gene interaction information from the STRING database. E. Frequency of mutations in DDR pathway-related genes in PDAC patients within TCGA dataset. F. Correlation between TOPBP1 and other key DDR gene mutations in PDAC patients in TCGA dataset. Data are presented as mean ± s.e.m. P-values were obtained from unpaired t-tests. n.s., not significant; *p < 0.05; **p < 0.01; ***p < 0.001. Fig. S2. Effect of TOPBP1 knockdown on Patu8988 cell proliferation in vitro. A. Western blot analysis demonstrating successful knockdown of the TOPBP1 protein in Patu8988, Panc1, and BxPC3 cells. B. Signaling pathway enrichment analysis of Biological Processes (BP) in Patu8988 cells following TOPBP1 knockdown. Several representatives signaling pathways were found to be altered, including those related to the negative regulation of cell cycle processes, spindle organization. C. Effect of TOPBP1 knockdown in Patu8988 cells assessed via EdU staining. Percentage of EdU-positive cells and EdU fluorescence intensity were quantified, revealing slight differences between the TOPBP1 knockdown and control cells. D. Impact of TOPBP1 knockdown on cell apoptosis evaluated via flow cytometry (FACS). Proportions of Annevin V/7-AAD staining are shown. Although there were slight differences in apoptosis, these differences were not statistically significant. Fig. S3. Impact of pathway changes in PDAC cells treated with olaparib, TOPBP1 knockdown, or both in vitro. A–B. Genome enrichment analysis (GSEA) identification of key DDR signaling pathways in TOPBP1-knockdown Patu8988 and BxPC3 cells. The representative DDR signaling pathways, namely base excision repair and non-homologous end joining, exhibited similar changing trends in both Patu8988 and BXPC3 cells following TOPBP1 knockdown. C. Gene set enrichment analysis (GSEA) identification of the top five representative signaling pathways in the BRCA1 non-mutation and PARP insensitivity group. D. Differences in signaling pathway enrichment between the BRCA1 non-mutation and PARP-insensitivity group were analyzed using Gene Ontology (GO) Biological Processes (BP). E–F. Signaling pathway enrichment compared through KEGG analysis and Reactome enrichment analysis in BXPC3 and Patu8988 cells treated with olaparib, TOPBP1 knockdown, or both. G. Interaction between endogenous TOPBP1 and P53 in Patu8988 cells examined by co-immunoprecipitation using an anti-TOPBP1 antibody or control mouse IgG. Fig. S4. Olaparib attenuates pancreatic tumor growth in subcutaneous xenograft PDAC mouse models. A–B. Tumor volumes of Topbp1-knockdown cells decreased significantly after treatment with olaparib compared to the control group on day 21. C–D. Weight change of mice within the model. No noticeable systemic toxicity is observed, as assessed by weight loss. Data are presented as mean ± s.e.m. P-values were obtained from unpaired t-tests. n.s., not significant; *p < 0.05. Fig. S5. Different sensitivities of PDAC cell lines and primary cells with various expression levels of TOPBP1 and other main DDR-related genes towards olaparib and AZD6738. A. IC50 values determined by the CCK8 assay demonstrate the drug sensitivity of PDAC cell lines to AZD6738. B. Western blot analysis of TOPBP1 and other proteins encoded by main DDR-related genes in primary PDAC cells. C. IC50 values determined by the CCK8 assay demonstrate the drug sensitivity of PDAC primary cells to olaparib. D. IC50 values determined by the CCK8 assay demonstrate the drug sensitivity of PDAC primary cells to AZD6738.

## Data Availability

The datasets supporting the conclusions of this article can be obtained from the corresponding authors.
