# Supplementary material for: TOPBP1 as a potential predictive biomarker for enhanced combinatorial efficacy of olaparib and AZD6738 in PDAC
Source: Cell Biosci. 2025 Feb 7;15:17. doi: 10.1186/s13578-025-01350-9 (PMC11806807; doi:10.1186/s13578-025-01350-9)
Supplement: Supplementary file 1 — Supplementary Material 1. Fig. S1. TOPBP1 is closely associated with the DDR pathway. A–B. TOPBP1 expression in pan-cancer TCGA sequencing data assessed using independent or paired sample. C. TOPBP1 expression was higher in PDAC patients with progressive disease (PD) therapy outcomes compared to those with complete response (CR), stable disease (SD), or partial response (PR) therapy outcomes based on TCGA datasets (n = 179, p = 0.016). D. TOPBP1 gene interaction network based on gene–gene interaction information from the STRING database. E. Frequency of mutations in DDR pathway-related genes in PDAC patients within TCGA dataset. F. Correlation between TOPBP1 and other key DDR gene mutations in PDAC patients in TCGA dataset. Data are presented as mean ± s.e.m. P-values were obtained from unpaired t-tests. n.s., not significant; *p < 0.05; **p < 0.01; ***p < 0.001. Fig. S2. Effect of TOPBP1 knockdown on Patu8988 cell proliferation in vitro. A. Western blot analysis demonstrating successful knockdown of the TOPBP1 protein in Patu8988, Panc1, and BxPC3 cells. B. Signaling pathway enrichment analysis of Biological Processes (BP) in Patu8988 cells following TOPBP1 knockdown. Several representatives signaling pathways were found to be altered, including those related to the negative regulation of cell cycle processes, spindle organization. C. Effect of TOPBP1 knockdown in Patu8988 cells assessed via EdU staining. Percentage of EdU-positive cells and EdU fluorescence intensity were quantified, revealing slight differences between the TOPBP1 knockdown and control cells. D. Impact of TOPBP1 knockdown on cell apoptosis evaluated via flow cytometry (FACS). Proportions of Annevin V/7-AAD staining are shown. Although there were slight differences in apoptosis, these differences were not statistically significant. Fig. S3. Impact of pathway changes in PDAC cells treated with olaparib, TOPBP1 knockdown, or both in vitro. A–B. Genome enrichment analysis (GSEA) identification of key [file 13578_2025_1350_MOESM1_ESM.docx]

**Supplemental information**

**TOPBP1 as a Potential Predictive Biomarker for Enhanced Combinatorial Efficacy of Olaparib and AZD6738 in PDAC**

Supplemental information contains 5 supplemental figures and legends and raw data of Western blotting.


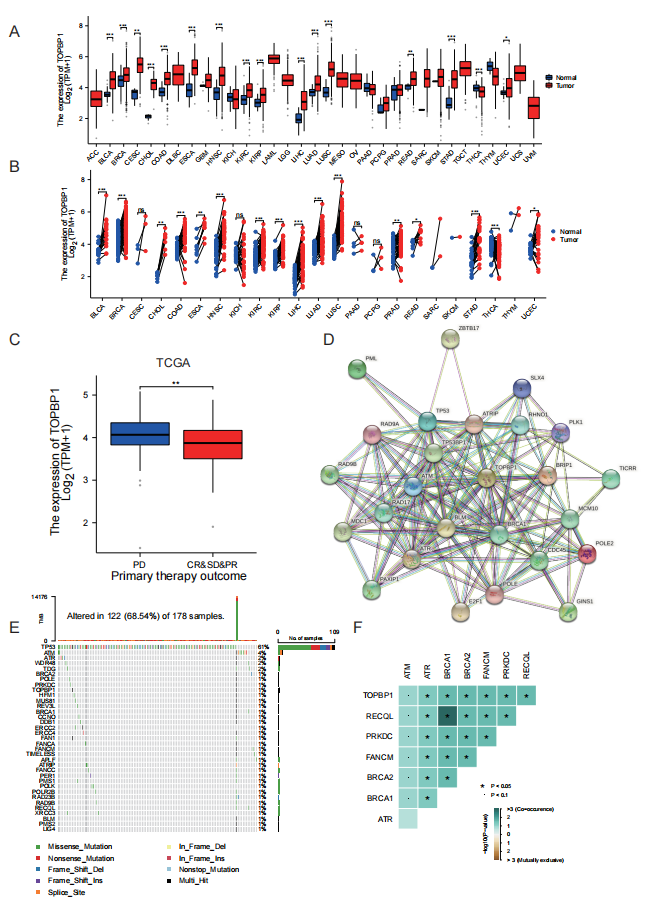
Fig. S1. TOPBP1 is closely associated with the DDR pathway. A–B. TOPBP1 expression in pan-cancer TCGA sequencing data assessed using independent or paired sample. C. TOPBP1 expression was higher in PDAC patients with progressive disease (PD) therapy outcomes compared to those with complete response (CR), stable disease (SD), or partial response (PR) therapy outcomes based on TCGA datasets (n = 179, p = 0.016). D. TOPBP1 gene interaction network based on gene–gene interaction information from the STRING database. E. Frequency of mutations in DDR pathway-related genes in PDAC patients within TCGA dataset. F. Correlation between TOPBP1 and other key DDR gene mutations in PDAC patients in TCGA dataset. Data are presented as mean ± s.e.m. P-values were obtained from unpaired t-tests. n.s., not significant; *, p < 0.05; **, p < 0.01; ***, p < 0.001.


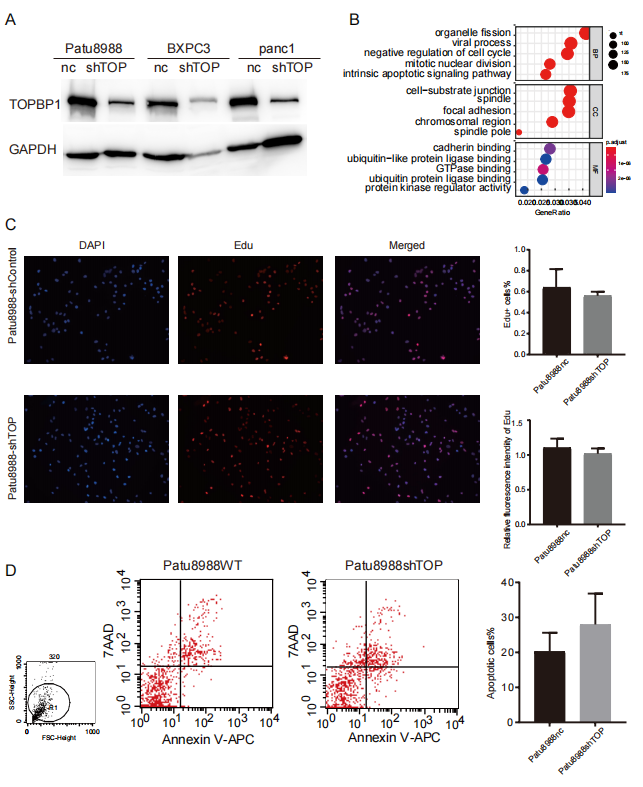
Fig. S2. Effect of TOPBP1 knockdown on Patu8988 cell proliferation in vitro. A. Western blot analysis demonstrating successful knockdown of the TOPBP1 protein in Patu8988, Panc1, and BxPC3 cells. B. Signaling pathway enrichment analysis of Biological Processes (BP) in Patu8988 cells following TOPBP1 knockdown. Several representatives signaling pathways were found to be altered, including those related to the negative regulation of cell cycle processes, spindle organization. C. Effect of TOPBP1 knockdown in Patu8988 cells assessed via EdU staining. Percentage of EdU-positive cells and EdU fluorescence intensity were quantified, revealing slight differences between the TOPBP1 knockdown and control cells. D. Impact of TOPBP1 knockdown on cell apoptosis evaluated via flow cytometry (FACS). Proportions of Annevin V/7-AAD staining are shown. Although there were slight differences in apoptosis, these differences were not statistically significant.


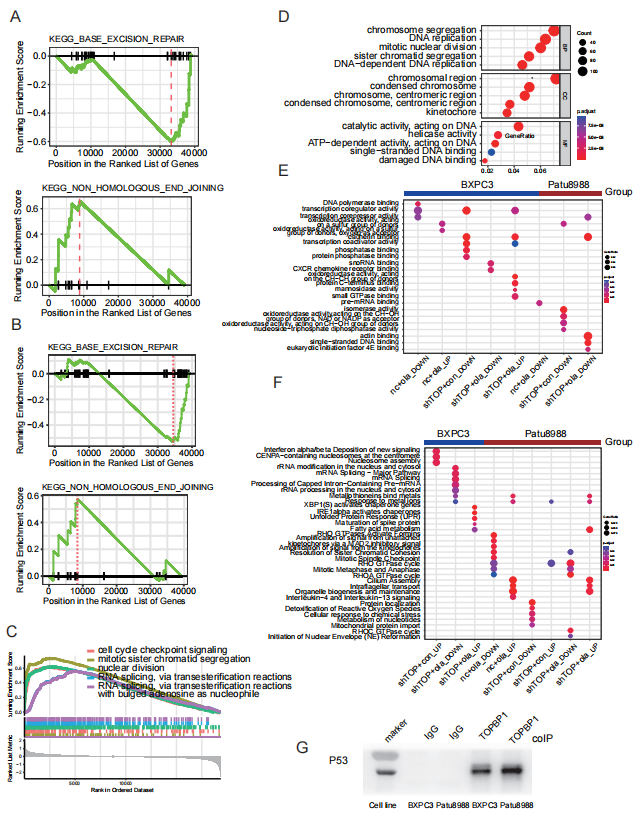
Fig. S3. Impact of pathway changes in PDAC cells treated with olaparib, TOPBP1 knockdown, or both in vitro. A–B. Genome enrichment analysis (GSEA) identification of key DDR signaling pathways in TOPBP1-knockdown Patu8988 and BxPC3 cells. The representative DDR signaling pathways, namely base excision repair and non-homologous end joining, exhibited similar changing trends in both Patu8988 and BXPC3 cells following TOPBP1 knockdown. C. Gene set enrichment analysis (GSEA) identification of the top five representative signaling pathways in the BRCA1 non-mutation and PARP insensitivity group. D. Differences in signaling pathway enrichment between the BRCA1 non-mutation and PARP-insensitivity group were analyzed using Gene Ontology (GO) Biological Processes (BP). E–F. Signaling pathway enrichment compared through KEGG analysis and Reactome enrichment analysis in BXPC3 and Patu8988 cells treated with olaparib, TOPBP1 knockdown, or both. G. Interaction between endogenous TOPBP1 and P53 in Patu8988 cells examined by co-immunoprecipitation using an anti-TOPBP1 antibody or control mouse IgG.


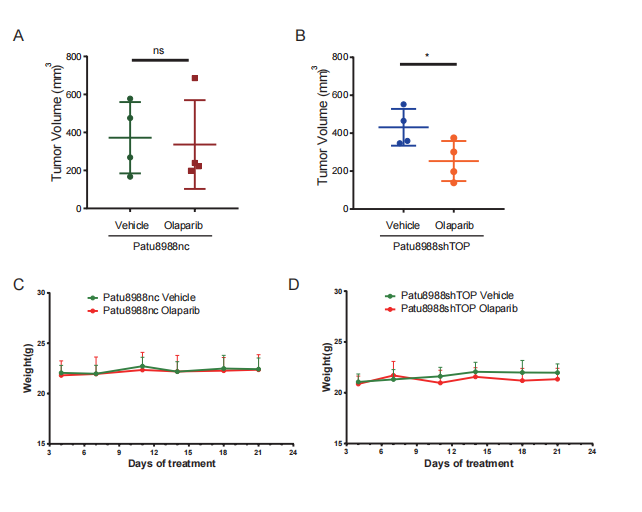
Fig. S4. Olaparib attenuates pancreatic tumor growth in subcutaneous xenograft PDAC mouse models. A–B. Tumor volumes of Topbp1-knockdown cells decreased significantly after treatment with olaparib compared to the control group on day 21. C–D. Weight change of mice within the model. No noticeable systemic toxicity is observed, as assessed by weight loss. Data are presented as mean ± s.e.m. P-values were obtained from unpaired t-tests. n.s., not significant; *, p < 0.05.


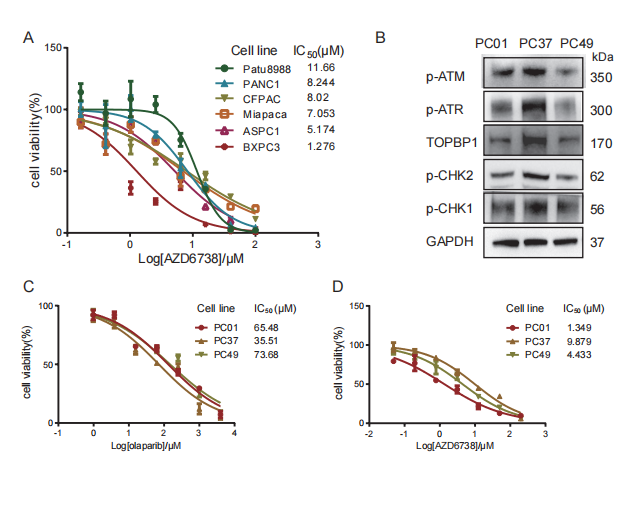
Fig. S5. Different sensitivities of PDAC cell lines and primary cells with various expression levels of TOPBP1 and other main DDR-related genes towards olaparib and AZD6738.

A. IC50 values determined by the CCK8 assay demonstrate the drug sensitivity of PDAC cell lines to AZD6738. B. Western blot analysis of TOPBP1 and other proteins encoded by main DDR-related genes in primary PDAC cells. C. IC50 values determined by the CCK8 assay demonstrate the drug sensitivity of PDAC primary cells to olaparib. D. IC50 values determined by the CCK8 assay demonstrate the drug sensitivity of PDAC primary cells to AZD6738.
